# A comparison of the functional results and costs of functional cast and volar-flexion ulnar deviation cast at 2-year follow-up in 105 patients aged 65 and older with dorsally displaced distal radius fracture: A randomized controlled trial

**DOI:** 10.1371/journal.pone.0283946

**Published:** 2023-04-06

**Authors:** Maarit Ax, Aleksi Reito, Teemu P. Hevonkorpi, Vili Palola, Juha Kukkonen, Toni Luokkala, Minna K. Laitinen, Antti P. Launonen, Ville M. Mattila

**Affiliations:** 1 Faculty of Medicine and Health Sciences, University of Tampere, Tampere, Finland; 2 Department of Orthopedics and Traumatology, Tampere University Hospital Tampere, Tampere, Finland; 3 Department of Surgery, Central Finland Central Hospital Nova, Jyväskylä, Finland; 4 Department of Orthopedics and Traumatology, Satakunta Central Hospital, Pori, Finland; 5 Department of Orthopedics and Traumatology, Helsinki University Hospital, Helsinki, Finland; Massachusetts General Hospital, UNITED STATES

## Abstract

**Background and purpose:**

Non-operative treatment is the most common treatment option for older patients with distal radius fracture (DRF). Traditionally, wrists have been placed in volar-flexion and ulnar deviation position (VFUDC). In recent years, there has been a trend towards using a functional position cast (FC). However, long-term results for these different casting positions are lacking.

**Patients and methods:**

This randomized, controlled, prospective study evaluates the functional results and costs of the 2 casting positions in patients 65 and older with DRF. Primary end point in this study was Patient-Reported Wrist Evaluation (PRWE) at 24 months, and secondary end points were cost-effectiveness of treatment, health-related quality of life measurement (15D), short version of Disabilities of arm, shoulder and hand score (QuickDASH), and VAS at 24 months. The trial was registered in ClinicalTrials.gov (NCT02894983, https://clinicaltrials.gov/ct2/show/NCT02894983).

**Results:**

We enrolled 105 patients, of which 81 (77%) continued until 24-month follow-up. 8 patients (18%) were operated in the VFUDC group and 4 (11%) in the FC group. Patients in the VFUDC group also received more frequent physical therapy. The difference in PRWE score between the VFUDC and FC groups at 24 months was -4.31. The difference in the cost of treatment per patient was €590. Both findings favored FC.

**Interpretation:**

We found a slight, but consistent difference in the functional results between groups. These results suggest that VFUDC is not superior to FC when treating Colles’ type DRF. Cost analysis revealed overall costs in the VFUDC group are nearly double those in the FC group, mostly due to more physical therapy, additional visits to hospital, and additional examinations. Therefore, we recommend FC in older patients with Colles’ type DRF.

## Introduction

Distal radius fracture (DRF) is the most common fracture among adults. Individuals 65 years and older are at the highest risk of sustaining DRF, Court-Brown & Caesar [1]. The most common type of DRF is a dorsally angulated fracture, the Colles’ fracture. Over the years, various immobilization methods for DRF have been introduced but it remains unclear which is the optimal method, Handoll & Madhok [2]; Caruso et al. [3]; Gamba et al. [4]; Gupta [5].

The volar-flexion and ulnar deviation cast (VFUDC) is still widely used as the primary immobilization method for DRF. It has been suggested, that in the VFUDC method fracture reduction is maintained using the principle of ligamentotaxis, Agee [6]. In the functional position cast (FC) method, the wrist is immobilized in a neutral position to ease the mobilization of the fingers and to reduce the risk of contractures during the immobilization period. In our previous study that compared the 12-month results of these cast positions, we found minor differences, including fewer complications and better functional results, favoring the FC method over VFUDC. We also found that fewer patients in the FC group required operative treatment compared to the VFUDC group, Raittio et al. [7]. Thus, we hypothesized that VFUDC leads to higher treatment costs in patients aged 65 and older.

The treatment of DRF causes not only disability to individual patients but also significant costs for health care systems globally. The cost of treatment varies greatly between countries, ranging from €533 in Spain to €4 028 in Sweden, Borgström et al. [8]. Determining the optimal way to treat these fractures could significantly reduce costs. The aim of this study was therefore to compare the 2-year functional results and costs of the VFUDC and FC immobilization methods for dorsally displaced DRFs in patients aged 65 years and older.

## Patients and methods

### Study design

This pragmatic, randomized controlled, multicenter trial compared two commonly used below elbow cast positions (VFUDC and FC) in patients 65 years and older who had sustained a dorsally angulated distal radius fracture. The patients for this study were enrolled at three large emergency hospitals in Finland: Tampere University Hospital (Tampere), Central Finland Central Hospital (Jyväskylä), and Satakunta Central Hospital (Pori). The study protocol has been published elsewhere, Raittio et al. [9], and the study has been registered in Clinicaltrials.gov. After publication of the protocol (S1 Protocol), a few changes were made to the study design and methods. These changes are outlined and described in detail in (S1 Appendix). The Regional Ethics Committee of the Expert Responsibility area of Tampere University Hospital approved the trial (ref ETL R16035). The patients were recruited between July 2016 and May 2017 and followed up until May 2019.

### Enrollment and randomization

All patients aged 65 years or older with a Colles’ type DRF and a sufficient reduction result were eligible for randomization (criteria presented in S2 Appendix). Due to the pragmatic nature of the study, there were no definitive restrictions for a sufficient reduction and non-operative treatment, but we used national care guidelines as a benchmark for decision making: dorsal angulation < 15 degrees, radial shortening < 3 mm, volar angulation < 15 degrees, intra-articular step < 1 mm, and radial inclination > 15 degrees, National Guideline [10]. All patients received written and oral information about the trial and gave their informed consent to participate in the trial prior to enrollment. The patients with unsatisfactory reduction results were excluded from the study.

The patients were randomized to either the VFUDC or FC group using a random number matrix in block allocation fashion with a 1 to 1 ratio. The blocks were stratified by age (65 to 74 and 75 and older) and whether the fracture was intra- or extra-articular. The randomization allocations were sealed in consecutively numbered envelopes that were situated in the emergency rooms of the participating hospitals.

### Procedure

Following enrollment, closed reduction of the fracture was performed under local anesthesia and the wrist was placed in a volar-flexion and ulnar deviation cast or a functional position cast according to the randomization. No fluoroscopy was used but x-rays were taken following reduction. All casts were non-circumferential, below-elbow plaster casts. Each hospital had an example cast to show the desired position (images of casts provided in S1 Fig).

### Follow-up

Initially, the aim was that the casting period would last for 5 weeks. The cast was only changed if there were problems with the cast or patient has symptoms related to the cast or if the patient was operated. Follow-up appointments were arranged at 1, 2, and 5 weeks after sustaining the fracture. For some patients, this arrangement was compromised due to loss of reduction and subsequent operative treatment before the end of the intended casting period. Operative treatment was performed after discussion with the patient and consultation with upper extremity surgeons. Guided physiotherapy was introduced when required.

Each participating hospital organized an additional follow-up visit at 3 months after the fracture. For research purposes, the questionnaires completed at 12 and 24 months were collected by Tampere University Hospital personnel. The complete assessment table of the protocol is provided in the study protocol, Raittio et al. [9].

### Outcome measures

The primary outcome was the Patient-Reported Wrist Evaluation (PRWE) score between the study groups at 24 months. Secondary outcomes were differences in means in the short version of Disabilities of arm, shoulder and hand, Quick-DASH score (qDASH, gives a scale from 0 to 100), visual analogue scale of pain (VAS, scale from 0 to 100 mm), health-related quality of life (15D, scale from 0 to 1), number of complications, number of surgical interventions, number of cast changes, and differences in the costs of the treatment. The PRWE is a 15-item questionnaire designed to measure wrist pain and disability in activities of daily living, MacDermid et al. [11]. The PRWE results are reported from 0 to 100, where 0 is the best suggesting no disabilities at all. The minimum clinically important difference (MCID) in PRWE was considered to be 11 points, Walenkamp et al. [12]. The 15D is a self-administrated questionnaire for measuring health-related quality of life (HRQoL). It is based on a set of Finnish population-based preferences, Sintonen [13]. The MCID in the 15D was considered to be 0.015 points.

In power calculations, we determined the required sample size per group to be 40 patients. This calculation was done by using the primary outcome variable (PRWE) with 95% confidence interval, power of 0.95, and SD of 14. Thus, to have enough statistical power, 40 patients in both groups had to complete 2-year follow-up.

### Cost analysis

We evaluated the costs between the two treatment groups by recording the number of cost-inducing contacts patients had with healthcare professionals. We recorded the number of visits to the hospital due to symptoms in the fractured wrist, surgery when needed, the number of physical therapy guidance visits, and possible additional examinations, such as plain radiographs, electroneuromyography (ENMG) examinations, and possible other imaging. Normal control visits of the study protocol were not included in the analysis since there was no variation between groups due to the protocol. Cost per contact was evaluated using pricing calculated by Tampere University Hospital Diagnosis-Related Group (DRG) for each year.

Quality-adjusted life years (QALYs) were calculated using the 15D at 24 months.

### Statistical analysis

The analysis included all patients who completed the questionnaires at 24-month follow-up. Differences between the two casting groups in PRWE score, qDASH score, VAS, 15D, and the effect of fracture in the dominant hand on PRWE were analyzed using t-test. Linear regression was used to estimate adjusted group difference in each outcome variable. The covariates used were age, gender, articular/non-articular fracture, and hand dominance. Regression diagnostics revealed there was an issue with residuals based on the QQ-plot. Therefore, we excluded one major outlier. This improved the explained variance from 4.5% to 9%. There were still concerns about the modeling assumptions. We augmented our adjusted analyses with a cumulative probability model which only assumed monotonicity of dependent variable, meaning it is insensitive to outliers. The predicted values from this model were reported and compared to the unadjusted and adjusted analyses. All analyses were performed with Rstudio 4 (Version 2021.09.1, Boston, US).

## Results

### Participants

From July 2016 to May 2017, a total of 105 patients from three Finnish hospitals were recruited into the trial and randomized to 2 groups. In total, 55 patients were assigned to the VFUDC group and 50 to the FC group The patients were then observed for 24 months until May 2019. The baseline characteristics of the recruited patients are presented in Table 1. The mean age of the patients at the time of recruitment was 73.5 years and 92 (88%) of the patients were female.

**Table 1:** The baseline characteristics of patients in the Volar Flexion Ulnar Deviation Cast (VFUDC) and Functional Cast (FC) groups.

Characteristic	VFUDC	FC
Number of patients	55	50
Age, mean (range)	72.6 (65–94)	74.5 (65–89)
Sex (female/male)	48/7 (87%/13%)	44/6 (88%/12%)
Dominant hand (right/left)	50/5	47/2
Fracture side (right/left)	27/28	18/32
Fracture in dominant hand	28 (51%)	18 (36%)
Extra-/intra-articular fractures	42/13	32/18

**Fig 1:**
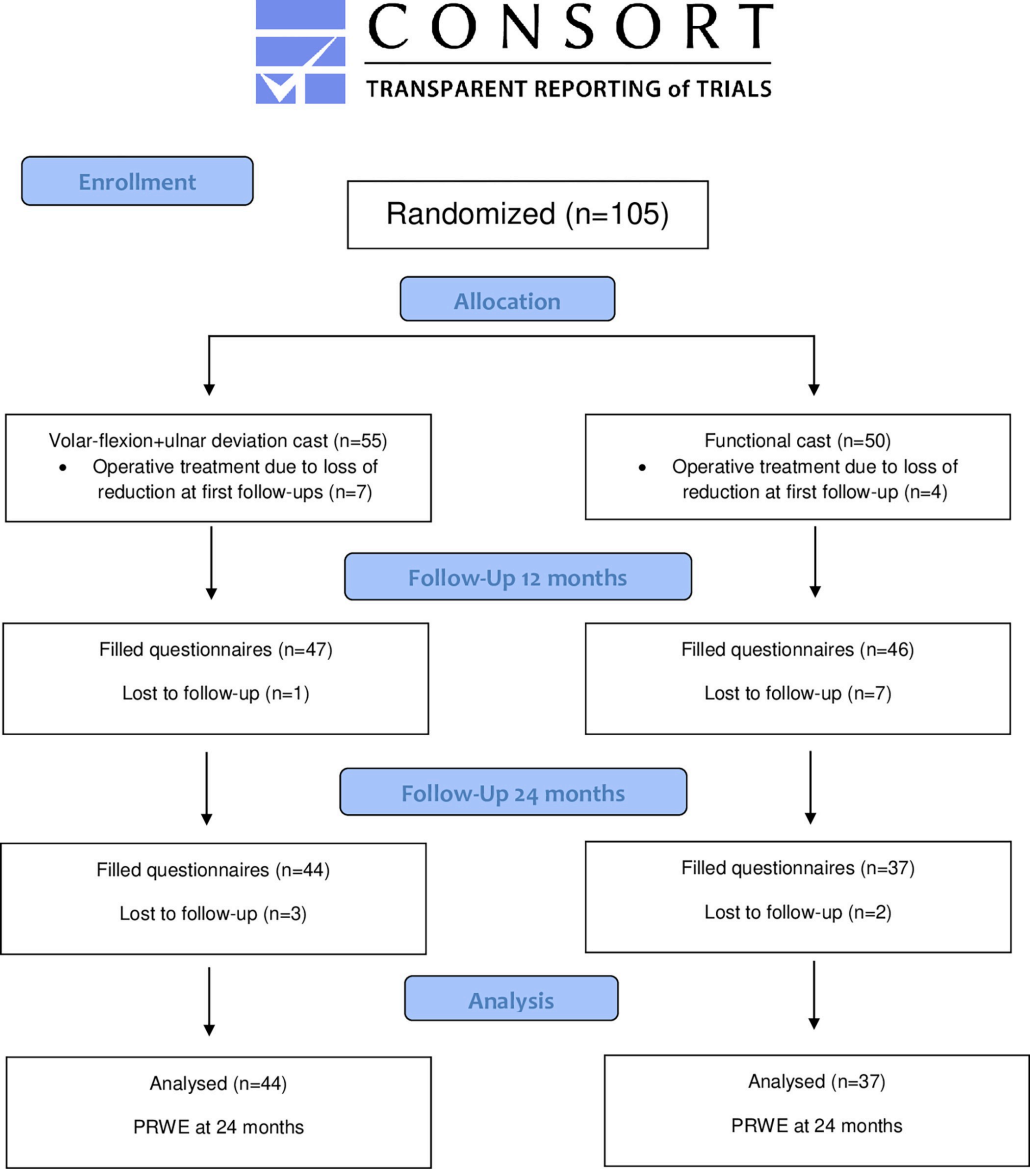
Randomization of patients and inclusion in the final analysis.

At 12-months, 19 patients had been lost to follow-up and a total of 24 patients did not return the questionnaires at 24-months following recruitment. Thus, 81 patients were included in the analysis (Fig 1). 80% of the recruited patients in the VFUDC and 74% of the patients in FC group were included in the final analysis. There were no major differences between the groups in the baseline characteristics in the final analysis, these are presented in (S1 Table).

### Outcomes at 24 months

#### PROMs and health-related quality of life

The outcome measures, PRWE, VAS, Quick-DASH, and 15D were recorded at 3, 12, and 24 months after fracture. These results are presented in Table 2.

**Table 2:** Analysis of the outcome measures for DRF at 3, 12, and 24 months in the VFUDC and FC treatment groups.

Evaluation	VFUDC (n)	SD	FC (n)	SD	Difference of means (95% confidence interval)
**PRWE **					
3 months	36.0 (47)	23.7	30.6 (44)	20.7	-5.4 (-14.7–3.9)
12 months	20.4 (47)	21.7	15.5 (39)	15.4	-4.9 (-13.1–3.4)
24 months	15.6 (44)	20.8	11.3 (37)	17.2	-4.3 (-12.8–4.2)
**Quick-DASH **					
3 months	34.7 (44)	23.5	31.3 (38)	22.2	-3.4 (-13.5–6.7)
12 months	20 (46)	19.3	17.2 (38)	15.1	-2.8 (-10.4–4.9)
24 months	17.9 (44)	22.4	15.1 (37)	19.5	-2.8 (-12.2–6.5)
**15 D **					
3 months	0.89 (46)	0.11	0.87 (43)	0.12	-0.02 (-0.07–0.03)
12 months	0.89 (47)	0.11	0.87 (39)	0.12	-0.01 (-0.06–0.03)
24 months	0.89 (39)	0.13	0.87 (28)	0.12	-0.02 (-0.08–0.05)
**VAS (mm) **					
3 months	24.3 (50)	20.7	21.0 (44)	18.4	-3.3 (-11.4–4.7)
12 months	15.6 (46)	20.3	12.6 (38)	15.2	-3.0 (-10.9–4.9)
24 months	14.0 (42)	21.9	10.2 (34)	16.8	-3.8 (-12.9–5.24)

VFUDC = Volar Flexion Ulnar Deviation Cast, FC = Functional Cast, PRWE = Patient-Reported Wrist Evaluation, Quick-DASH = short version of Disabilities of Arm, Shoulder and Hand, 15D = self-administrated questionnaire for measuring health-related quality of life, VAS = Visual Analogue Scale of pain

There was a slight difference between the groups concerning whether the patient had the fracture in the dominant hand (51% in VFUDC vs 36% in FC group). This did not, however, seem to influence the functional results between the groups. The difference between fracture in the dominant and non-dominant hand in PRWE scores at 24 months was 1.6 (95% CI -10 to 7).

In a linear regression model, the adjusted difference between groups in PRWE at 24 months was -5.3 points (95% CI -13 to 2.2) in favor of FC when adjusted for age, sex, articular/non-articular fracture, and hand dominancy. This did not exceed the MCID. The adjusted difference between groups in 15D at 24 months was -0.006 points (95% CI -0.06 to 0.05). The results from the cumulative probability model are presented in Table 3. These results were in line with the other analyses.

**Table 3:** Predicted outcomes from the cumulative probability model for PRWE at 24 months for each combination of baseline variables. FC = Functional Cast group, VFUDC = Volar-Flexion Ulnar Deviation Cast group.

	ARTICULAR FRACTURE		NON-ARTICULAR FRACTURE	
	Dominant hand	Non-dominant hand	Dominant hand	Non-dominant hand
**FC **				
**Female **				
70	11.8	10.0	9.4	7.9
75	13.8	11.8	11.2	9.4
80	16.1	13.8	13.1	11.1
**Male **				
70	11.4	9.6	9.1	7.6
75	13.4	11.4	10.8	9.1
80	15.6	13.4	12.7	10.7
**VFUDC **				
**Female **				
70	18.1	15.6	14.8	12.7
75	20.8	18.1	17.2	14.8
80	23.8	20.8	19.9	17.2
**Male **				
70	17.6	15.1	14.4	12.2
75	20.2	17.5	16.7	14.3
80	23.1	20.2	19.3	16.6

The number of cast changes was recorded per patient’s report. The number of cast changes was higher in the VFUDC group (25 cast changes) than in the FC group (15). The number of casts per patient was 1.57 in the VFUDC group and 1.41 in the FC group.

### Cost analysis

The costs of treatment consisted of additional visits to the hospital, imaging and other examinations, physical therapy, and operative treatment. The mean overall cost of treatment per patient in the VFUDC group was €1 307 and €717 in the FC group. Thus, the costs per patient of VFUDC were €590 (82%) higher than the costs of FC. The difference in median costs was €-156 (95% CI -303 to -9), again in favor of FC. The difference in means in a linear regression model of 15D at 2 years was 0.006 points (95% CI -0,05 to 0,06) and did not reach the MCID. Based on 15D at 2 years, FC produced 0.012 more QALYs compared to VFUDC.

The mean number of additional visits to hospital per patient was 1.98 in the VFUDC group and 1.64 in the FC group. Additional visits were 21% more frequent in VFUDC than in FC group. The reason for additional visits in the hospital was contact from the patient due to pain, pressure sores and/or numbness of fingers. Further examinations and procedures were done as needed according to the evaluation by a consultant surgeon. The overall costs of these visits and associated additional imaging or other examinations related to the fracture were €443 per patient in the VFUDC group and €358 per patient in the FC group. The rate of patients who had received physical therapy after 24 months was 57% (25) in the VFUDC group and 37% (14) in the FC group. The total number of physical therapy contacts was 224 in the VFUDC group and 63 in the FC group. The mean number of physical therapy contacts was 10.4 for the VFUDC and 4.5 for the FC group and the median were 6 and 3, respectively. The costs of physical therapy were €17 472 in the VFUDC group and €4 914 euros in the FC group. The cost of physical therapy per patient was €397 in the VFUDC and €133 in the FC group.

A total of 6 operations were performed in the VFUDC group due to loss of reduction within the intended casting period. All operations were performed using an anatomic volar locking plate (VLP). In the FC group, 4 operations were performed. No corrective osteotomies were performed in the FC group, whereas 2 osteotomies were performed in the VFUDC group during the two-year follow-up period. Two patients in the VFUDC group had electroneuromyography performed after the casting period ended, but none of the patients needed surgery due to carpal tunnel syndrome and no carpal tunnel release operations were performed during the follow-up period. The cost of a surgical operation for fracture was €2 092, and the cost of a corrective osteotomy was €3 997 euros. The cost of operations per patient in the VFUDC group was €467 and €226 in the FC group. Delayed surgery increased the cost of operative care in the VFUDC group by €182 per patient. Excluding the cost of surgeries and only considering additional costs due to physical therapy and additional visits and examinations, the overall cost of treatment per patient was 840 euros per patient in the VFUDC and 490 euros in the FC group, a difference of 71% between groups.

## Discussion

In this randomized, controlled, multicenter study, we found a small, imprecise but consistent, difference between groups in all primary and secondary functional results (PRWE, 15D QD, and VAS) favoring FC. The primary outcome, PRWE at 24 months, showed a difference of 5.37 points. However, this did not reach the predetermined MCID of 11 points. Surprisingly, the fracture being in the dominant or non-dominant hand did not seem to influence PRWE at 24 months or predict outcome in the cumulative probability model. Overall, the rate of operative treatment, physical therapy and additional visits to the outpatient clinic, and additional imaging and other examinations was higher in the VFUDC group. This caused the mean cost of treatment to be substantially higher in the VFUDC group than in the FC group. The increase in QALY was produced with lower costs compared to VFUDC. In other words, VFUDC produces inferior QALYs at a higher cost. It should also be noted that QALYs tend to measure the cost burden of the overall course of the illness/treatment and may not therefore be fully applicable in assessing the individual burden of disease, especially in the early phases of the treatment. In our study, not only were the overall costs of visits and treatment higher, but patients in the VFUDC group more often reported feeling pain (26% vs 9%, p = 0 .04) and stiffness (9% vs 0%, p = 0.06) to yes/no questions compared to the FC group at 3 months, Raittio et al. [7] even though the other outcome measures (PRWE, Quick-DAS, 15D, VAS) did not show MCID. In addition, 8 patients in the VFUDC group had more than 2 cast changes compared to 4 in the FC group, Raittio et al. [7]. This transient effect might resemble the burden of the selected treatment, especially in the acute phase (<3 months).

Current protocols concerning the non-operative treatment of DRF seem to vary greatly between countries, hospitals, and even surgeons. Different casting methods and positions have been used based on fracture morphology, and some authors have also advocated different approaches, for example, Colles’ fractures compared to Smith fractures, van Leeuwen et al. [14]. In addition, similar types of fractures have been treated with a variety of options regarding casting position, cast material, length (above vs below elbow cast), method (circular or splint cast), and the duration of immobilization, Delft et al. [15]. Even sugar-tong and active dynamic splints have been suggested as means to prevent pronation-supination movement in the forearm, Çamur et al. [16]; Sarmiento & Latta [17]. The best casting position for the wrist, the duration of immobilization, and the best material for the cast are yet to be determined, Handoll & Madhok [2]. However, a circular cast or an above-elbow cast does not seem to be necessary, Wik et al. [18]; Caruso et al. [3]; Okamura et al. [19]. Some studies have also suggested that dorsal flexion seems to be superior to palmar flexion in functional results Gupta [5]; Grle et al., [20]; Rajan [21]. Based on our results, functional cast position should be used.

Most patients 65 years and older with a DRF are suitable for non-operative treatment and the functional results of operative and non-operative treatment are similar in the older population, Stephens et al. [22]; Luokkala et al. [23]. In the present study, some patients were allocated to operative treatment due to a loss of reduction during follow-up. This was expected as the patients and the reduction of the fracture were monitored during the casting period. As the population has aged and the treatment possibilities have developed, the costs of healthcare have risen markedly over the past decades, WHO: Global Spending on Health: Weathering the Storm [24]. According to the World Health Organization, global spending on healthcare reached 10% of global GDP in 2018. Therefore, there is an urgent need to prioritize resources and to find new ways to reduce the costs of existing treatments without lowering the quality of the treatment. In the United States, operative treatment using volar locking plate has been shown to be markedly more expensive than non-operative treatment, with costs of $3 784 and $718, respectively, Shauver et al. [25]. In a Swedish study, the costs for non-operative treatment were $137 and $1 698 for plate fixation, Navarro et al. [26]. In a recent Norwegian study comparing operative treatment to non-operative in the treatment of displaced DRFs in patients 65 years and older, healthcare provider costs were €3 859 in the operative group and €2 056 in the non-operative group. With a willingness to pay €27 500 per QALY, there was a 45% chance of the operative treatment being effective. The authors concluded that operative treatment was not cost-effective from a healthcare perspective, Hassellund et al. [27]. The cost effectiveness of different operative treatment options in the older population have also been studied. These studies concluded that of the three commonly used operative treatment options, VLP, external fixation, and percutaneous pinning with K-wires, VLP was the most expensive Saving et al. [28]; Shauver et al. [25].

According to a Finnish study, the incidence rate of distal radius fracture among individuals aged 60 years and older in Oulu, Finland, was 585 per 100 000 people per year, Flinkkilä et al. [29]. In 2017, the number of people aged 60 years and older in Finland was approximately 1 179 000. This means that the estimated number of distal radius fractures among people aged 60 years and older is approximately 6 900 per year. In Finland alone, the difference in costs between these different conservative treatment options (€590) in the older population would be more than 4 million euros annually. It is unclear whether patients benefit from supervised physiotherapy compared to independent exercises. However, it seems that after DRF treated operatively or conservatively, supervised physiotherapy does not lead to superior outcomes when compared with independent exercises, Quadlbauer et al. [30]; Bruder et al. [31]. In our study, the patients in the VFUDC group received physiotherapy more often than the patients in the FC group, resulting in substantially higher costs. In the VFUDC group, supervised physiotherapy made up 30% of the overall costs, whereas it made up 18.5% in the FC group. Despite the lower cost, the functional results seemed to be consistently better in the FC group. As guided physiotherapy does not seem to have a positive effect on functional results, the routine referring of patients to guided physiotherapy should be carefully evaluated. Instead, the need for physiotherapy should be evaluated by shared decision-making with the patient to identify those individuals who would benefit most from physiotherapy.

### Strengths and limitations

In this study, we enrolled 105 patients. The drop-out rate at 12 months was 18% (19) and 23% (24) at 24 months. This was a higher rate than we expected, and the number of patients in the FC group was therefore lower than expected in the power analysis. However, the drop-out rate did not exceed 30% for long-term follow-up and we may conclude that the risk of bias has not increased. The sample size we used does not, however, permit convincing conclusions for infrequent outcomes, such as the difference in the rate of complications between the two studied interventions, to be made. Additionally, the SD of our primary outcome, PRWE score at 24 months, was larger than anticipated and utilized in a priori power calculation. However, the functional results remained consistent regardless of this.

## Conclusions

We found a slight, but consistent difference in functional results between the VFUDC and FC groups. Our results suggest that FC produces non-inferior results compared to VFUDC in the treatment of Colles’ type of DRFs at 2 years with fewer problems and unnecessary healthcare provider visits, and less treatment-related burden. Cost analysis revealed costs were nearly double in the VFUDC group compared to the FC group, mostly due to more frequent physical therapy, surgery, and additional visits to hospital and additional examinations. Regardless of the more frequent physiotherapy visits, the VFUDC group had consistently slightly inferior functional results. In conclusion, we recommend the use of FC in older patients with Colles’ type DRF. Moreover, careful critical consideration should be given to routine physiotherapy guidance among older patients with distal radius fracture.

## Supporting information

S1 Checklist CONSORT 2010 checklist of information to include when reporting a randomised trial. (DOC)

S1 Appendix Description of changes to the published protocol. (DOCX)

S2 Appendix Inclusion and exclusion criteria for the trial. (DOCX)

S1 Fig Photos of the casts used in the trial. (DOCX)

S1 Table Baseline data of patients included in the final analysis. (XLSX)

S1 Protocol Initial trial protocol. (DOC)
