# Supplementary material for: A comparison of the functional results and costs of functional cast and volar-flexion ulnar deviation cast at 2-year follow-up in 105 patients aged 65 and older with dorsally displaced distal radius fracture: A randomized controlled trial
Source: PLoS One. 2023 Apr 6;18(4):e0283946. doi: 10.1371/journal.pone.0283946 (PMC10079055; doi:10.1371/journal.pone.0283946)
Supplement: S1 Fig — (DOCX) [file pone.0283946.s004.docx]

Pictures of the functional cast and volar-flexion ulnar deviation cast


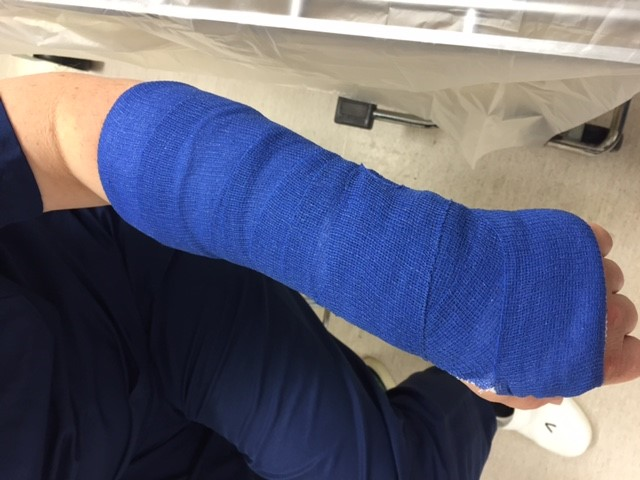


Picture 1. Volar-flexion and ulnar deviation cast, above view


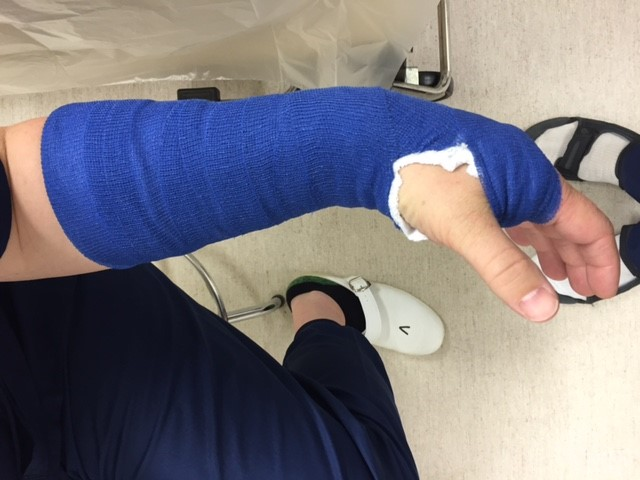


Picture 2. Volar-flexion and ulnar deviation cast, side view


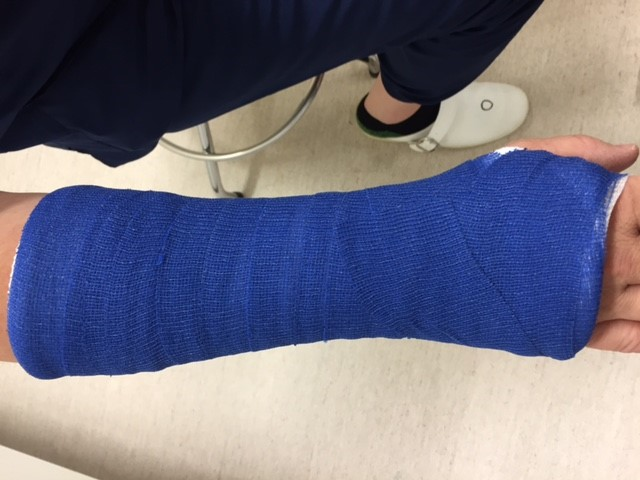


Picture 3. Functional cast, above view


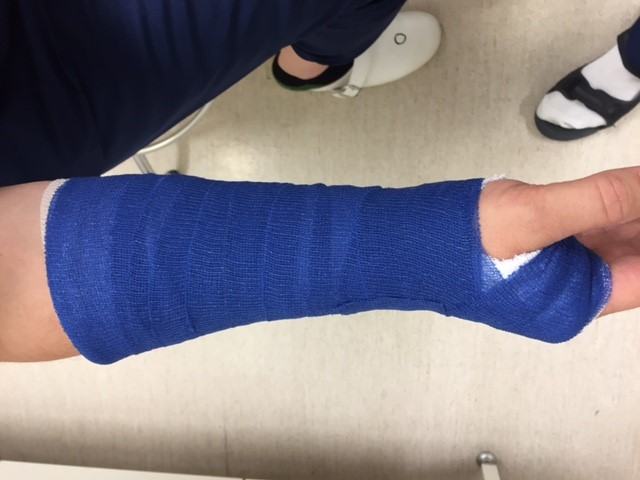


Picture 4. Functional cast, side view
